# Risk factors for self-reported medication adherence in community-dwelling older patients with multimorbidity and polypharmacy: a multicenter cross-sectional study

**DOI:** 10.1186/s12877-023-03768-7

**Published:** 2023-02-06

**Authors:** Jiaming Liu, Yongpei Yu, Suying Yan, Yan Zeng, Su Su, Tiantian He, Zimin Wang, Qian Ding, Ruixue Zhang, Wenchao Li, Xin Wang, Lan Zhang, Xiaolin Yue

**Affiliations:** 1 Department of Pharmacy, Xuanwu Hospital, Capital Medical University, Beijing, China; 2 Department of Biostatistics, Peking University Clinical Research Institution, Beijing, China; 3 Department of Medical Affairs, Xuanwu Hospital, Capital Medical University, Beijing, China; 4 Ethics Committee, Xuanwu Hospital, Capital Medical University, Beijing, China

**Keywords:** Medication adherence, Potentially inappropriate medication, Multimorbidity, Polypharmacy, Older adults

## Abstract

**Background:**

Medication nonadherence is a significant public health problem as it contributes to poor clinical outcomes and increased healthcare costs. Older patients with multimorbidity and polypharmacy often have low medication adherence. These patients also have a high prevalence of potentially inappropriate medication (PIM) use.

**Aim:**

To explore risk factors related to medication nonadherence in older patients with multimorbidity and polypharmacy and examine the association between medication nonadherence and PIM use.

**Method:**

A multicenter cross-sectional study was conducted from May to December 2019 in 16 tertiary hospitals from 12 provinces and cities in China. Data were collected from outpatients 65 years or older with multimorbidity and polypharmacy. The PIMs were evaluated using the 2019 Beers Criteria. Self-reported medication adherence was assessed using the Visual Analog Scale (VAS).

**Results:**

A total of 773 outpatients were recruited. The prevalence of medication nonadherence was 31.8%. In the univariate analysis, nonadherence was significantly associated with sex, cognitive impairment, stroke, visiting the same physicians, self-administration of medication, the percentage of drug costs ≥ 10% of the medical expenses, and PIMs for the alimentary tract and metabolism. In the multivariate analysis, the results almost paralleled those of the univariate associations. Notably, the use of PIM was significantly associated with medication adherence.

**Conclusion:**

Several factors that influence medication adherence were identified. Targeted interventions can be implemented to improve medication adherence, such as encouraging self-administering medications and reducing medication expenses.

## Introduction

According to the World Health Organization (WHO), the world population of people aged 60 years and older will reach 2.1 billion in 2050 [1]. China is currently experiencing an accelerated period of population aging. There will be 400 million Chinese citizens 65 years or older by 2030 [2]. With an aging population, the prevalence of multimorbidity (defined as having two or more chronic medical conditions) is likely to increase rapidly [3]. Older adults with multimorbidity require long-term treatment. Multiple medications are often prescribed simultaneously to these patients, leading to polypharmacy [4, 5]. Polypharmacy is usually defined as taking five or more medications simultaneously, although no consensus has been reached on the exact cutoff number [6, 7]. In the United States, 39% of adults over 65 take five or more daily medications [8]. A retrospective study in China reported that 15.9% of people aged over 65 years of age had multimorbidities [9]. Another Chinese study reported that 65.2% of patients aged over 60 years of age took five or more daily medications [10].

Medication adherence refers to the ability of patients to follow their medication schedule as prescribed by their healthcare provider. Medication adherence remains a significant challenge in older patients with chronic diseases [11, 12]. Nonadherence can lead to treatment failure, increased hospital readmissions related to medications, additional medical/surgical procedures, and excess healthcare costs [11, 13]. It can also result in mortality [14].

The risk factors for medication nonadherence are complex and relate to patients and drugs. Patient-related factors include sex [15], age [13], physical inactivity [16], cognitive function [5, 17, 18], marital status [13, 16], education level [19], caregivers [20], alcohol consumption [16], and multimorbidity [16]. Drug-related factors include the number of medications [18], copays [18], drug costs [5, 13], and adverse drug reactions [5, 17, 18]. The use of potentially inappropriate medications (PIM) in older patients is widespread. PIM use is associated with adverse drug events (ADE) and affects medication adherence [5, 21, 22].

Few studies have been conducted on medication nonadherence in older patients with multimorbidity and polypharmacy [23, 24]. This study aimed to evaluate the associations between patient characteristics, drug-related factors, and medication adherence in community-dwelling older patients with polypharmacy and chronic diseases. Furthermore, this study also examined the association between medication non-adherence and PIM use.

## Methods

### Study design and participants

The study was a multicenter cross-sectional study conducted from May to December 2019 in 16 tertiary hospitals in 12 provinces and cities in China. A convenience sampling method was used to recruit study participants. The inclusion criteria were patients: (1) 65 years or older, (2) diagnosed with two or more diseases, and at least one of the following five chronic diseases (hypertension, diabetes mellitus, coronary heart disease, dyslipidemia, and stroke), (3) taking five or more medications (traditional Chinese medicines were not included) for a period of ≥ 6 months. Patients who could not participate or did not complete the interview were excluded.

The project was approved by the Research Ethics Committee of the Xuanwu Hospital of Capital Medical University (approval number [2018] 022, June 25, 2018). It was registered by the Research Ethics Committees of the participating hospitals.

### Sample size calculation

Based on the previous studies, we assumed that the proportion (P) of polypharmacy was 65.3% [9]. A minimum of 710 participants were required (the width of the 95% confidence interval of the proportion of polypharmacy < 8%, a dropout rate of 20%). The sample size was calculated using PASS 16. Fifty participants were recruited from each hospital, as 16 tertiary hospitals participated in this study. Finally, 800 participants were included in the study.

### Medication adherence

Medication adherence was assessed using the visual analog scale (VAS), a self-reported measure of medication adherence. VAS asks individuals to mark a line at the point along a continuum (0–100), showing how well they followed physician orders in the last four weeks [25]. For example, 0 refers to the patient not following the physician’s instructions, and 100 means that the patient strictly follows physician orders. A score below 80 is considered medication nonadherence [26]. The interviews with cognitively impaired patients were conducted in the presence of their caregivers whenever possible to minimize the misreporting of VAS.

### Data collection and standardization

Data were collected from face-to-face interviews using a standardized questionnaire. The following data were collected: age, sex, marital status, living condition, educational attainment, height, weight, smoking and alcohol consumption, medication, ability to self-administer medications, medical insurance, comorbidities, history of ADE, physician visits, hospital admissions, and percentage of drug costs within medical expenses. Height and weight were used to calculate Body Mass Index (BMI, kg/m^2^). Educational attainment was divided into low (below the middle school, < 9 years) and high (≥ 9 years) ranges. The Mini-Cog test was used to detect cognitive impairment [27]. Cognitive impairment was when the Mini-Cog scores ≤ 2.

The use of PIM was evaluated using the 2019 Beers criteria developed by the American Geriatrics Society [28]. The following five categories (tables) are in the criteria: Category A: Medications that are potentially inappropriate in most older adults; Category B: Medications that are potentially inappropriate in older adults with certain conditions; Category C: Medications that should be used with caution; Category D: Potentially clinically important drug-drug interaction and Category E: Medications that should be avoided or have their dosage reduced based on kidney function. Since serum creatinine values were not available, the PIMs listed in Category E were not evaluated. Two researchers assessed PIM use, and a third researcher was invited to resolve the discrepancy through discussions.

Diagnoses were recorded by the 10th revision of the International Statistical Classification of Diseases and Related Health Problems (ICD 10). Medication was classified using the WHO Anatomical Therapeutic Chemical (ATC) classification (2016 version).

### Statistical analysis

Descriptive statistics are expressed as medians and interquartile ranges (IQR) for quantitative variables and as frequencies and proportions for categorical variables. Univariate analysis was performed with Student’s* t*-test for normally distributed quantitative variables. The Wilcoxon rank-sum test was used for nonnormally distributed quantitative variables. Chi-square tests were used for categorical variables in the univariate analysis. Adjusted odds ratios (aOR) and 95% confidence intervals (95%CI) were estimated using a multivariate logistic regression model. *P* < 0.05 was considered statistically significant. Significant risk factors associated with medication nonadherence in univariate analysis were analyzed in the multivariable logistic regression analyses. Given the exploratory purpose of this study, the type-I error was not adjusted for multiple tests. Data were analyzed using SAS9.4 (Cary, NC, USA).

## Results

### Patients’ demographic and clinical characteristics

A total of 800 patients completed face-to-face interviews, and 773 were included in the analysis. Fifteen had missing information and 12 did not meet the requirements of comorbidity and polypharmacy. The median age of the participants was 73.7 (69.1, 79.6) years, and 58.9% (455/773) were men. Most of the patients were married and lived with family members. A total of 164 patients (21.2%) had cognitive impairment. The median number of comorbidities was 5 (4, 6). Patients were taking a median number of 6 (5, 7) medications. Hypertension was the most prevalent chronic disease, at 88.4% (683/773). This corresponded to the highest percentage of patients taking medication for the cardiovascular system, 98.7% (763/773). Detailed information is shown in Table 1.

**Table 1:** Patients’ characteristics, medication information, PIMs prevalence, medication with PIM use, and VAS scale for medication adherence

Variable (*n* = 773)	Item	Total, n (%)
Age, years	Median (IQR)	73.7(69.1, 79.6)
	65–74	431(55.8%)
	≥ 75	342(44.2%)
Gender	Male	455(58.9%)
	Female	318(41.1%)
Marital status	Married	653(84.5%)
	Single/divorced/widowed	120(15.5%)
Living condition	Living with family members	665(86.0%)
	Alone	108(14.0%)
Education attainment	Low^a^	602(77.9%)
	High^b^	171(22.1%)
BMI (kg/m^2^)	Median (IQR)	24.3(22.0, 26.7)
Smoking	Yes	256(33.1%)
	No	517(66.9%)
Alcohol drinking	Yes	82(10.6%)
	No	691(89.4%)
Cognitive impairment	Yes	164(21.2%)
	No	609(78.8%)
Hospital admissions within the last year	Yes	448(58.0%)
	No	325(42.0%)
Comorbidities	Number, median (IQR)	5.0(4.0, 6.0)
	Hypertension	683(88.4%)
	Coronary heart disease	492(63.7%)
	Diabetes mellitus	422(54.6%)
	Dyslipidemia	372(48.1%)
	Stroke	273(35.3%)
Visiting the same physicians	Yes	388(50.2%)
	No	385(49.8%)
Number of prescribers	1–2	607(78.5%)
	3 and above	166(21.5%)
Medications	Number, Median (IQR)	6.0(5.0, 7.0)
	5–7	660(85.4%)
	8–9	92(11.9%)
	> 10	21(2.7%)
ATC Classification^c^	Cardiovascular system	763(98.7%)
	Alimentary tract and metabolism	679(87.8%)
	Blood and blood-forming organs	316(40.9%)
	Nervous system	182(23.5%)
Ability to self-administer medications	Yes	683(88.4%)
	No	90(11.6%)
History of ADE	Yes	140(18.1%)
	No	633(81.9%)
Medical insurance	Yes	733(94.8%)
	No	40(5.2%)
% of drug costs of medical expenses	< 10%	321(41.5%)
	≥ 10%	452(58.5%)
PIM use		479(62.0%)
Medications category with PIMs	Blood and blood-forming organs	214(27.7%)
	Cardiovascular system	183(23.7%)
	Alimentary tract and metabolism	86(11.1%)
	Nervous system	83(10.7%)
	Genito urinary system and sex hormones	13(1.7%)
	Musculo-skeletal system	6(0.8%)
	Systemic hormonal preparations, excl. sex hormones and insulins	4(0.5%)
	Antineoplastic and immunomodulating agents	1(0.1%)
Medication adherence (VAS scale)	Median (IQR)	88.0(65.0, 100.0)
	0–19	5(0.7%)
	20–39	34(4.4%)
	40–59	114(14.7%)
	60–79	88(11.4%)
	80–100	532(68.8%)

*IQR* Interquartile range, *BMI* Body mass index, *ADE* Adverse drug event, *PIM* Potentially inappropriate medication, *VAS* Visual analogue scale, *ATC* Anatomical Therapeutic Chemical

^a^Primary school and below were included

^b^Middle school and above were included

^c^Only the categories of medications used more than 10% were listed

### The prevalence and analysis of PIM use

The prevalence of PIMs was 62.0% (479/773). Patients mainly took PIMs from the ATC medication categories of blood and blood-forming organs (27.7%, 214/773), cardiovascular system (23.7%, 183/773), and the alimentary tract and metabolism (11.1%, 83/773).

### Analysis of medication adherence and factors associated with medication adherence

**Fig. 1:**
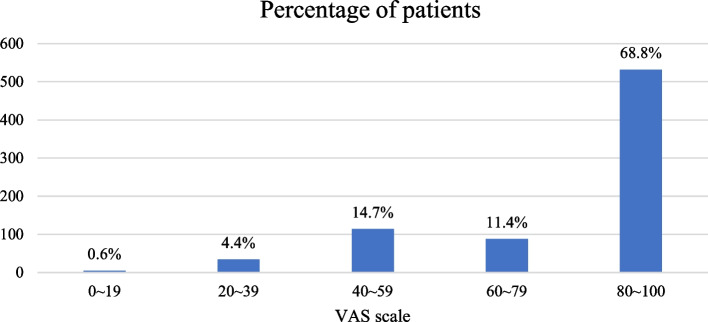
Histogram of the percentage of patients stratified by 20% of the VAS

Among study participants, 532 (68.8%) patients had a VAS score above 80, indicating medication adherence (Table 1). The median VAS score was 88.0 (65.0, 100.0). Figure 1 shows the VAS score distribution stratified by 20%.

Table 2 shows the univariate analysis comparing patient demographic and clinical characteristics between the medication adherent and nonadherent groups. There were significant differences in sex (*P* = 0.002), cognitive impairment (*P* < 0.001), stroke (*P* = 0.003), visiting the same physicians (*P* = 0.013), self-administering medications (*P* < 0.001), the percentage of drug costs ≥ 10% of the medical expenses (*P* = 0.002), and taking PIMs from the alimentary tract and metabolism ATC category (*P* = 0.02) between the two groups.

**Table 2:** Univariate analysis for association of adherence with patients’ characteristics and medication information (*n* = 773)

Variables	Item	VAS Score < 80 (*n* = 241)	VAS Score ≥ 80 (*n* = 532)	*P*
Age, years	Median (IQR)	73.3(68.6,80.0)	74.0(69.1,79.6)	0.455
	65–74	137(56.9)	294(55.3)	0.681
	≥ 75	104(43.2)	238(44.7)	
Gender	Male	122(50.6)	333(62.6)	**0.002**
	Female	119(49.4)	199(37.4)	
Marital status	Married	198(82.2)	455(85.5)	0.231
	Single/divorced/widowed	43(17.8)	77(14.5)	
Living condition	Living with family members	200(83.0)	465(87.4)	0.101
	Alone	41(17.0)	67(12.6)	
Education attainment	Low	196(81.3)	406(76.3)	0.120
	High	45(18.7)	126(23.7)	
BMI (kg/m^2^)	Median (IQR)	24.5(22.3,26.7)	24.3(22.0,26.7)	0.651
Smoking	Yes	69(28.6)	187(35.2)	0.074
	No	172(71.4)	345(64.9)	
Alcohol drinking	Yes	27(11.2)	55(10.3)	0.718
	No	214(88.8)	477(89.7)	
Cognitive impairment	Yes	73(30.3)	91(17.1)	** < 0.001**
	No	168(69.7)	441(82.9)	
Hospital admissions within the last year	Yes	148(61.4)	300(56.4)	0.190
	No	93(38.6)	232(43.6)	
Comorbidities	Number, Median (IQR)	5.0(4.0,6.0)	5.0(4.0,6.0)	0.311
	Hypertension	211(87.6)	472(88.7)	0.639
	Coronary heart disease	146(60.6)	346(65.0)	0.233
	Diabetes mellitus	129(53.5)	293(55.1)	0.689
	Dyslipidemia	123(51.0)	249(46.8)	0.275
	Stroke	67(27.8)	206(38.7)	**0.003**
Visiting the same physicians	Yes	105(43.6)	283(53.2)	**0.013**
	No	136(56.4)	249(46.8)	
Number of prescribers	1–2 physicians	189(78.4)	418(78.6)	0.963
	3 and above physicians	52(21.6)	114(21.4)	
Medications	Number, Median (IQR)	6.0(5.0,7.0)	6.0(5.0,7.0)	0.617
	5–7	207(85.9)	453(85.2)	0.076
	8–9	32(13.3)	60(11.3)	
	> 10	2(0.8)	19(3.6)	
	Cardiovascular system	235(97.5)	528(99.3)	0.079
	Alimentary tract and metabolism	212(88.0)	467(87.8)	0.942
	Blood and blood forming organs	97(40.3)	219(41.2)	0.810
	Nervous system	61(25.3)	121(22.7)	0.436
Ability to self-administer medications	Yes	199(82.6)	484(91.0)	** < 0.001**
	No	42(17.4)	48(9.0)	
History of ADE	Yes	48(19.9)	92(17.3)	0.380
	No	193(80.1)	440(82.7)	
Medical insurance	Yes	231(95.9)	502(94.4)	0.386
	No	10(4.2)	30(5.6)	
% of drug costs of medical expenses	< 10%	80(33.2)	241(45.3)	**0.002**
	≥ 10%	161(66.8)	291(54.7)	
PIM use		140(58.1)	339(63.7)	0.135
Medications category with PIMs	Blood and blood forming organs	6(2.5)	7(1.3)	0.241
	Cardiovascular system	44(18.3)	101(19.0)	0.810
	Alimentary tract and metabolism	63(26.1)	184(34.6)	**0.02**
	Nervous system	32(13.3)	48(9.0)	0.072
	Genito urinary system and sex hormones	1(0.4)	6(1.1)	0.445
	Musculo-skeletal system	2(0.8)	4(0.8)	1.000
	Systemic hormonal preparations, excl. sex hormones and insulins	2(0.8)	0(0.0)	0.097
	Antineoplastic and immunomodulating agents	1(0.4)	0(0.0)	0.312

*IQR* Interquartile ranges, *BMI* Body mass index, *ADE* adverse drug event, *PIMs* Potentially inappropriate medications, *VAS* visual analogue scale

Table 3 shows the results of the multivariate analysis of factors of medication nonadherence. Medication nonadherence was significantly associated with sex (aOR0.65, 95%CI 0.46–0.92, *P* = 0.015), cognitive impairment (aOR1.84, 95%CI 1.26–2.69, *P* = 0.002), the number of comorbidities (aOR1.13 95%CI 1.03–1.24, *P* = 0.011), stroke (aOR 0.44, 95%CI 0.30–0.65, *P* < 0.0001), the % of drug costs ≥ 10% of the medical expenses (OR1.51 95%CI 1.07–2.14 *p* = 0.020), ability to self-administer medications (aOR2.28, 95%CI 1.40–3.71, *P* = 0.001), and PIM use (aOR 0.71, 95%CI 0.50–0.99, *P* = 0.041).

**Table 3:** Multivariate analysis for the association of medication nonadherence with patients' characteristics and medication information (*n* = 773)

Variable	Item	aOR (95%CI)	*P*
Age, years	65–74 vs. ≥ 75	1.06(0.76,1.49)	0.731
Gender	Male vs. Female	0.65(0.46,0.92)	**0.015**
Living condition	Living with family members vs. Alone	0.66(0.42,1.04)	0.074
Alcohol drinking	Yes vs. No	1.39(0.81,2.39)	0.230
Cognitive impairment	Yes vs. No	1.84(1.26,2.69)	**0.002**
Comorbidities, number		1.13(1.03,1.24)	**0.011**
Hypertension	Yes vs. No	0.79(0.47,1.31)	0.354
Coronary heart disease	Yes vs. No	0.75(0.52,1.08)	0.125
Diabetes mellitus	Yes vs. No	0.79(0.55,1.12)	0.187
Dyslipidemia	Yes vs. No	1.05(0.74,1.50)	0.780
Stroke	Yes vs. No	0.44(0.30,0.65)	** < 0.0001**
Visiting the same physicians	Yes vs. No	0.74(0.53,1.04)	0.079
% of drug costs of medical expenses	≥ 10% vs. < 10%	1.51(1.07,2.14)	**0.020**
Medications, number		0.99(0.86,1.13)	0.844
Ability to self-administer medication	No vs. Yes	2.28(1.40,3.71)	**0.001**
History of ADE	Yes vs. No	1.13(0.74,1.73)	0.563
PIM use	Yes vs. No	0.71(0.50,0.99)	**0.041**

*aOR* Adjusted odds ratio, *CI* Confidence interval, *ADE* Adverse drug event, *PIMs* Potentially inappropriate medications

## Discussion

The study is one of the few studies that investigate the prevalence and risk factors of medication adherence in community-dwelling older adults with comorbidity and polypharmacy. Nonadherence to medications is common throughout the world. Only 50% of older patients who suffered from chronic diseases followed the treatment recommendations, and the rate was even lower in Asia, according to the 2013 WHO report [29]. The medication adherence rate in our study was 68.82%, higher than in similar studies conducted in China and other Asian countries, with reported rates of 32.6–45% [13, 23, 30, 31]. The differences are related to the study population and the adherence assessment tools. The present study population was older adults who lived in cities and were more likely to receive high-quality medical services from tertiary hospitals. In this study, we used the VAS scale to assess medication adherence. As a widely accepted self-reporting measure in medication adherence, the VAS scale has high levels of concordance with other adherence measures [32]. It has been validated in patients with chronic diseases or who need long-term medication therapies [26, 33]. The VAS scale is simple and easy to implement into the workflow to capture adherence information in a large population cohort. However, self-reporting has disadvantages, as patients are known to overestimate their level of adherence [34].

PIMs are medications whose potential risks outweigh their benefits [21, 35]. Several screening tools and criteria help identify PIM in older adults, and the American Geriatrics Society (AGS) Beers criteria are the most widely used and cited tool [5, 21, 36]. The prevalence of PIMs varies between 14.1–64.8% in the older adult population in China [37–39]. The prevalence of PIM use in this study was 62.0%. PIM prescribing increases significantly among older patients with polypharmacy [40]. Study subjects had a median of five comorbidities and were prescribed a median of six medications, which contributed to the higher rate of PIM use. The lack of awareness among physicians about PIM could be another contributing factor.

In the univariate analysis, medication adherence was not associated with age and educational status. However, in other studies, age and higher education status had better adherence [19, 41]. More female patients reported nonadherence in our research. Women generally play an essential role with the primary responsibility to care for other family members in the household. They may neglect to care for themselves and take their medications [42]. Cognitive impairment was associated with nonadherence, supported by previous studies [16, 17]. Patients with cognitive impairment have difficulty understanding medication instructions and remembering routes of administration [5, 18]. Visiting the same physicians regularly was associated with medication adherence. When patients saw the same physicians regularly, the physicians were updated about the changes in their condition. Medications could be appropriately adjusted, and ADE could be promptly managed, leading to improved medication adherence [43].

Stroke patients reported higher medication adherence than patients with other chronic diseases in this study, consistent with a previous study [44]. Stroke often leads to reduced cognitive function and self-care ability [42]. Patients or their family members may have paid more attention to medications due to the presence of stroke disabilities compared to patients with hypertension or diabetes, who usually do not have severe symptoms or disabilities [45]. The inability to take medications independently adversely affected medication adherence in this study. The support of a family caregiver is strongly correlated with medication adherence [45, 46]. Most of the patients in this study had health insurance. However, more than 50% of the patients had a percentage of drug costs that exceeded 10% of medical expenses, suggesting that the burden of the cost of medication was heavy [47]. Like other studies, medication costs negatively affected medication adherence [13, 48].

The results of the multivariate regression analysis almost paralleled those of the univariate associations. However, it should be noted that the number of comorbidities was linked to non-adherence. Previous studies demonstrate that complex disease conditions, polypharmacy, and PIM prescribing negatively impact medication adherence [32, 48, 49]. We hypothesized that PIM use could negatively affect medication adherence in our population. However, the results of this study indicate the opposite. A Japanese study showed that the use of PIM by older patients was significantly associated with self-reported medication adherence [23]. The reason may be related to the study population. Almost 90% of the study subjects could self-administer their medications. Patients who can self-administer their medications may have been educated and proactively monitored by physicians or pharmacists to prevent ADE or worsening outcomes [23]. Furthermore, the study subjects were motivated to take medications, as shown by the high median VAS score of 88. Moreover, the history of ADE, which was not strictly defined as events caused by PIM, was not associated with adherence in this study. Although some factors, such as sex, cognitive status, and comorbidities, cannot be changed, other factors, such as medication expenses and self-administering medications can improve medication adherence [50].

## Limitations

Our study has several limitations. First, the study population was older patients who visited tertiary hospitals in cities. The study sample may not represent older patients living in rural areas or visiting community hospitals. Further studies are needed to recruit older patients from different regions and hospitals. Second, it is common for adults in China to use traditional Chinese medicines. However, we did not assess these medications as they cannot be standardized, and the Beers criteria do not cover them. Finally, VAS is a self-reported method used to assess medication adherence. VAS overestimates adherence compared to direct measures such as pill counting or blood drug concentration testing. In contrast, lower VAS scores could be obtained from cognitively impaired individuals due to misreporting.

## Conclusion

Older adults with comorbidity and polypharmacy had good medication adherence and a high frequency of PIM use. Several factors influenced medication adherence, including cognitive status, medication expenses, ability to self-administer medications, and PIM use. Targeted interventions based on this study can be implemented to improve medication adherence.

## Data Availability

All data generated or analysed during this study are included in this published article.

## Abbreviations

PIM: Potentially inappropriate medication
VAS: Visual Analog Scale
WHO: World Health Organization
ADE: Adverse drug events
BMI: Body Mass Index
ICD: International Statistical Classification of Diseases and Related Health Problems
ATC: Anatomical Therapeutic Chemical
IQR: Interquartile ranges
aOR: Adjusted odds ratios
CI: Confidence intervals
AGS: American Geriatrics Society
